# Faster Adaptation in Smaller Populations: Counterintuitive Evolution of HIV during Childhood Infection

**DOI:** 10.1371/journal.pcbi.1004694

**Published:** 2016-01-07

**Authors:** Jayna Raghwani, Samir Bhatt, Oliver G. Pybus

**Affiliations:** Department of Zoology, University of Oxford, South Parks Road, Oxford, United Kingdom; University of New South Wales, AUSTRALIA

## Abstract

Analysis of HIV-1 gene sequences sampled longitudinally from infected individuals can reveal the evolutionary dynamics that underlie associations between disease outcome and viral genetic diversity and divergence. Here we extend a statistical framework to estimate rates of viral molecular adaptation by considering sampling error when computing nucleotide site-frequencies. This is particularly beneficial when analyzing viral sequences from within-host viral infections if the number of sequences per time point is limited. To demonstrate the utility of this approach, we apply our method to a cohort of 24 patients infected with HIV-1 at birth. Our approach finds that viral adaptation arising from recurrent positive natural selection is associated with the rate of HIV-1 disease progression, in contrast to previous analyses of these data that found no significant association. Most surprisingly, we discover a strong negative correlation between viral population size and the rate of viral adaptation, the opposite of that predicted by standard molecular evolutionary theory. We argue that this observation is most likely due to the existence of a confounding third variable, namely variation in selective pressure among hosts. A conceptual non-linear model of virus adaptation that incorporates the two opposing effects of host immunity on the virus population can explain this counterintuitive result.

## Author Summary

Since some common approaches to the study of molecular adaptation may not be optimal for answering questions regarding within-host virus evolution, we have developed an alternative approach that estimates an absolute rate of molecular adaptation from serially-sampled viral populations. Here, we extend this framework to include sampling error when estimating the rate of adaptation, which is an important addition when analyzing historical data sets obtained in the pre-HAART era, for which the number of sequences per time point is often limited. We applied this extended method to a cohort of 24 pediatric HIV-1 patients and discovered that viral adaptation is strongly associated with the rate of disease progression, which is in contrast to previous analyses of these data that did not find a significant association. Strikingly, this results in a negative relationship between the rate of viral adaptation and viral population size, which is unexpected under standard micro-evolutionary models since larger populations are predicted to fix more mutations per unit time than smaller populations. Our findings indicate that the negative correlation is unlikely to be driven by relaxation of selective constraint, but instead by significant variation in host immune responses. Consequently, this supports a previously proposed non-linear model of viral adaptation in which host immunity imposes counteracting effects on population size and selection.

## Introduction

The molecular evolution and adaptation of the human immunodeficiency virus (HIV) within infected individuals is exceptionally fast. This evolution is generated by a combination of high rates of mutation and recombination, large population sizes and short generation times, and has important consequences for the outcome and treatment of HIV infection [1]. For example, HIV is able to persist within hosts by evading host humoral and T-cell immune responses through the repeated generation and fixation of immune escape mutations. In addition, the evolution of resistance to anti-viral drugs represents a significant problem in HIV treatment.

Several approaches have been taken to quantify and understand the dynamics of HIV molecular evolution during infection. Experimental estimates of the virus’ mutation rate suggests that it can generate ~1.4x10^-5^ mutations per nucleotide site per replication event [2]. Evolutionary analyses of HIV gene sequences sampled longitudinally during infection indicate that the nucleotide substitution rate of the virus is approximately constant but varies among genome regions, ranging from 10^−2^ to 10^−3^ substitutions per nucleotide site per year [3–5]. Positive natural selection during HIV infection has been typically inferred using dN/dS ratios [6, 7], as well as by methods based on allele frequency changes [7, 8], and these studies sometimes suggest that viral adaptation is associated with the time taken for disease symptoms to progress to AIDS [7, 8] or rate of immune escape [9]. However, the interpretation of dN/dS ratios obtained from within-host viral populations are not straightforward due to the presence of transient polymorphisms [10, 11] and therefore alternative approaches to studying viral adaptation are valuable. Williamson [12] introduced a method to estimate an absolute rate of viral molecular adaptation, and reported that the C2-V5 region of the HIV *env* gene undergoes approximately 3 adaptive fixations per year during infection.

To date, most studies of the evolutionary dynamics of HIV during infection have examined infection in adults and many are based on the same cohort of nine untreated patients [5, 12]. However, significantly different clinical features characterize HIV infection in children, including a faster rate of disease progression (i.e. AIDS symptoms occur earlier), substantially higher viremia (levels of HIV in the blood can exceed 100,000 RNA copies per ml in pediatric infection) and a slower decline in viremia after initial infection compared to adult infections [13]. The clinical course of HIV infection in children also varies by age of infection and transmission route and, because infection takes place in patients with a developing immune system, a large variation in immune responses among patients is observed [14–16]. Recently it has been shown that development of broadly neutralizing antibodies (bNAb) in HIV-infected infants occurs early in infection and is relatively common [17]. Moreover, in spite of the major role that HLA class I polymorphisms play in determining adult HIV disease progression, a recent study has found that HLA alleles that are protective for adult infections are comparatively weak in HIV-infected children [18].

To better understand the dynamics of viral adaptation during pediatric HIV infection, we estimate the rate of within-host viral adaptation among a cohort of 24 children that acquired the virus through perinatal transmission. For each patient *env* gene sequences were sampled over 2 to 4 years of infection and were complemented by clinical measurements of viral load and CD4+ T-cell counts. Importantly, these measurements enable us to test associations between viral adaptation and the rate of disease progression, and to explore the determinants of variation in viral adaptation rate among infections. For both HIV and hepatitis C virus infection it has been suggested that disease progression is associated with measures of viral genetic diversity and viral adaptation [7, 8, 11, 12, 19]. Interpretation of these associations is often limited by implicit assumptions of linear relationships among viral population size, diversity, adaptation, and immune selection.

It is likely that the majority of adaptive molecular evolution detected in HIV *env* sequences is the result of viral escape from humoral immune responses. The importance of humoral immunity for the long-term control of viremia is supported by the observation that depletion of B cells during human or primate infections leads to dramatic increases in viral load [20–22]. Although it is known that cytotoxic T-lymphocyte (CTL) responses also play an important role in restricting HIV replication, CTL escape mutations are likely few and each occur once during infection. In contrast, the idea that humoral responses drive reciprocal and recurrent adaptive selection during infection is supported experimentally [23], by genetic analysis [9] and by theoretical models [24].

To estimate absolute rates of molecular adaptive evolution during HIV infection we employ a statistical framework [25–27] that is based on the classic McDonald-Kreitman test for positive selection [28] and on subsequent work [12, 29]. Our approach has been specifically developed for rapidly evolving viruses and relaxes assumptions that are not reasonable for these populations: previous work has shown that methods related to the McDonald-Kreitman test can be made more robust when applied to viral populations by taking into account multiple mutations at a given site [26] and by relaxing the unrealistic assumption that all polymorphisms are selectively neutral [25]. However, a continuing weakness of the framework is that it does not account for sampling error when counting the number of polymorphic and fixed sites in the alignment. This is particularly important for data sets with small numbers (2 to 50) of sequences per time point, including the data investigated here. To address this problem, we extend the framework by introducing a probabilistic model that incorporates binomial sampling error when calculating mutational site frequencies. A more detailed summary of the theoretical background of our approach can be found in the Materials and Methods section.

Notably, we discover a strong negative correlation between viral population size and the rate of molecular adaptation, which is the opposite of the relationship predicted by standard models of molecular evolution (in which fixation rates are proportional to the product of population size, per capita mutation rate, and fixation probabilities; e.g. [30]). We suggest a simple non-linear model that incorporates the counteracting effects of host immune responses on viral adaptation during infection provides a parsimonious explanation for this observation.

## Materials and Methods

### Sequence and clinical data

The viral sequences investigated here come from a cohort of 24 HIV-infected children recruited between 1986 and 1992 for the New York City Perinatal Transmission study [31, 32]. Detailed information about sample collection and sequencing methods are given elsewhere [31, 32]. The infections were acquired at or very close to the time birth. A quarter of these patients received no treatment during the study, while the remaining patients were treated with Zidovudine and/or Didanosine for a part of the study (S1 Table). The sequences available for analysis represent approximately 360 nt of the V3 region of HIV-1 envelope gene (positions 6963–7328 relative to the HXB2 genome). A heteroduplex mobility assay (HMA) was used to screen PCR clones for sequence variants per time point, so sequences may be slightly more variable compared to a perfectly random sample [33, 34]. The sequences were manually aligned using Se-Al [33, 35]. We estimated rates of adaptation from viral gene sequences obtained at the first and last sampling times for each patient. The first sampling time was on average ~ 2 months after birth (range 0 to 7 months) and the last sampling time was on average ~ 25 months after birth (range 8 to 55 months). For the first time point, between 1 and 15 sequences were obtained per patient, while for the last time point 3 to 15 sequences were sampled per patient. Measures of viral load and CD4+ T-cell count were also available for each patient, at an average of 5 time points per patient. The mean CD4+ and log viral load per patient were calculated by linearly interpolating between each measured value, then calculating the average value of the resulting piecewise linear function between the first and last sampling times. Furthermore, each patient was placed into one of four disease progression categories based on the CD4+ T cell counts and a clinical diagnosis of AIDS (S1 Table). In order of increasing clinical severity these categories are (i) slow non-progressors (ii) moderate non-progressors, (iii) moderate progressors and (iv) rapid progressors. Interestingly, viral loads are not noticeably different during periods of anti-viral drug therapy (for more details see Table S1 in Carvajal-Rodriguez et al. [6]).

### Theoretical background

To estimate absolute rates of molecular adaptive evolution during HIV infection we employ a statistical framework [25–27] that has been developed specifically for rapidly evolving viruses. This approach is based on the classic McDonald-Kreitman test for positive selection [28] and on subsequent work [12, 29]. A brief introduction to the methodology is given here; further details of implementation and validation are provided in [25, 26].

**Fig 1:**
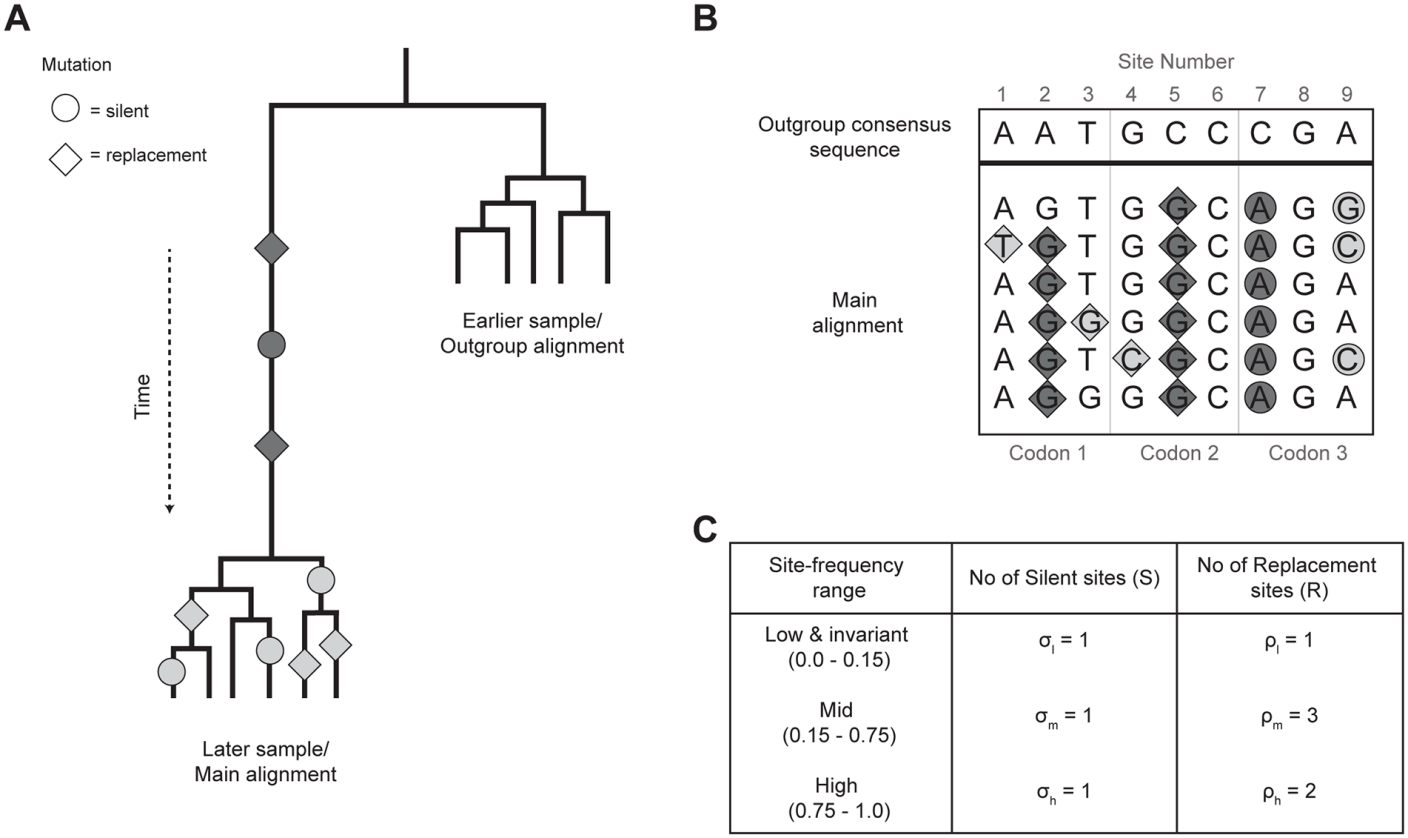
A schematic diagram that outlines the method used to estimate the rate of molecular adaptation in serially-sampled populations. (A) Viral sequences sampled from an earlier time point (the outgroup alignment) are compared with sequences sampled at a later time point (the main alignment). Mutations on the internal branch leading to the later sample (dark grey) represent nucleotide fixations, while all remaining mutations (light grey) correspond to polymorphisms in the later sample. Replacement (non-synonymous; diamonds) and silent (synonymous; circles) mutations are distinguished. (B) A consensus of the sequences from the earlier time point is used to identify whether fixations and polymorphisms are ancestral or derived. In this example, mutation has occurred in 7 out of 9 sites in the main alignment. (C) Nucleotide site-frequencies (i.e. the frequency of each mutation in the main alignment) are calculated and probabilistically assigned to three site-frequency ranges for both silent and replacement changes. Under neutral evolution, the ratio of replacement to silent changes in the mid site-frequency range, ρ_m_/ σ_m_, is expected to equal to the corresponding ratio in the high site-frequency range (ρ_h_/ σ_h_). Excess replacement changes in the high site-frequency range thus represent adaptive substitutions driven by positive selection (eq 2). Note that invariant sites in the alignment (i.e. sites 6 and 7 in panel B) are assigned as silent or replacement using the degeneracy of the genetic code (see S2 Table for details). Further, the site-frequency of invariant sites is probabilistically assigned using a Beta-binomial model (see Materials and Methods).

To infer natural selection, two sets of homologous gene sequences are required: a ‘main’ alignment and an ‘outgroup’ alignment. In the context of estimating adaptive evolution in rapidly evolving viruses, these two alignments correspond to the viral population being sampled at two different time points (Fig 1A). In the HIV-1 infected patients studied here, the main alignment comprises sequences from a later sampling time, while the outgroup alignment represents sequences from an earlier sampling time, during the acute phase of infection. Given the limited viral genetic diversity observed at the earlier sampling time, the outgroup alignment can be effectively replaced by a single consensus sequence (Fig 1B). In other words, sequences from the earlier time point represent the ancestral viral population. Sequences from the later sampling time are then compared to the consensus sequence from the first sampling time, and each nucleotide site is classified according to its observed frequency in the population (i.e. its derived site-frequency). We further infer whether the derived mutation represents a nonsynonymous or synonymous change with respect to the consensus sequence at the first sampling time (Fig 1C). To ensure consistency in terminology and equations with previous work [20] we refer to nonsynonymous mutations as ‘replacement’ and synonymous mutations as ‘silent’.

### Estimating site frequency

By comparing the main and ancestral alignments, each site in the main alignment is defined as invariant (no polymorphism and identical to the ancestral nucleotide), fixed (no polymorphism and different to the ancestral nucleotide), or polymorphic. If the site in the main alignment is polymorphic, then the ancestral alignment is used to define which nucleotides are ancestral and which are derived. Rules based on fractional counting are used when three or more nucleotides are present at a site. For example, if we observe a two-state polymorphic site in the main alignment that does not include the ancestral nucleotide, the most parsimonious explanation for this site is that an earlier fixation event occurred which was then followed by another mutation at the same site. The classic McDonald-Kreitman test (i.e. assuming the infinite sites model) would treat this site as a polymorphism, leading to an underestimation of the number of fixation events. In contrast the fractional counting method treats this site as equally representing both a fixation and a polymorphism. Further details about the counting algorithm can be found in Bhatt et al [26].

Suppose that the main alignment consists of *N* viral gene sequences, *K* nucleotides in length. If *D*_*i*_ denotes the number of derived nucleotides at site *i* in the main alignment, then the estimated frequency of the derived nucleotide at that site is simply *D*_*i*_/*N*. However, this estimate has a large binomial variance when sample size is small; the true frequency of a site that appears ‘fixed’ in a sample of 5 to 10 sequences may be considerably less than one. Similarly, a site that appears invariant in the main alignment (i.e. *D*_*i*_ = 0) may actually be polymorphic in the study population.

If *p*_*i*_ denotes the true frequency of the derived state at site *i*, then we model the probability of *p*_*i*_ given *N* and *D*_*i*_ using a Beta-Binomial Bayesian model. The ancestral (*N-D*_*i*_) and derived (*D*_*i*_) site-frequencies are dichotomous random variables for which the canonical likelihood function is the Binomial distribution. We model the prior distribution of the Binomial parameter *p*_*i*_ as a Beta[1,1] distribution (equivalent to a unit uniform distribution). The resulting normalized posterior distribution is therefore described by conjugacy as a Beta distribution with the form:

$$\text{P}\left({\text{p}}_{\text{i}}|\text{N},{\text{D}}_{\text{i}}\right)=\frac{\left(\text{N}+1\right)!}{\left(\text{N}-{\text{D}}_{\text{i}}\right)!{\text{D}}_{\text{i}}!}{\text{p}}_{\text{i}}{}^{{\text{D}}_{\text{i}}}{\left(1-{\text{p}}_{\text{i}}\right)}^{\text{N}-{\text{D}}_{\text{i}}}$$ (1)

The probability that *p*_*i*_ lies between the interval *u* and *v* is therefore an integral over the posterior Beta distribution within the range {*u*,*v*}:

$$\text{P}\left(\text{u}<{\text{p}}_{\text{i}}<\text{v}|\text{N},{\text{ D}}_{\text{i}}\right)=\;\frac{\left(\text{N}+1\right)!}{\left(\text{N}-{\text{D}}_{\text{i}}\right)!{\text{D}}_{\text{i}}!}\left[\int_{\text{u}}^{\text{v}}{\text{p}}_{\text{i}}{}^{{\text{D}}_{\text{i}}}{\left(1-{\text{ p}}_{\text{i}}\right)}^{\left(\text{N}-{\text{D}}_{\text{i}}\right)}\text{ dp}\right]$$ (2)

Hence the expected number of sites with a derived nucleotide frequency between *u* and *v* is

$${\hat{\text{f}}}_{\text{u},\text{v}}=\sum_{\text{i}=1}^{\text{K}}\text{P(u}<{\text{p}}_{\text{i}}<\text{v|N},{\text{D}}_{\text{i}})$$ (3)

The values *u* and *v* define a ‘site-frequency range’ that contains f_u,v_ sites. Since f_0,1_ = K the interval [0, 1] can be split into any number of non-overlapping site-frequency ranges. Note that this means that site frequencies are estimated for all sites, including invariant sites, not just for polymorphic sites.

The expected number of sites in each range can be calculated separately for silent (synonymous) and replacement (non-synonymous) sites (Fig 1C). Polymorphic and fixed sites in the main alignment are classified as silent or replacement by direct comparison with the ancestral alignment. Invariant sites are classified as silent, replacement, or undefined using a fractional approach based on the codon degeneracy inherent in the genetic code (see S2 Table).

If *ρ*_u,v_ and *σ*_u,v_ define the expected number of replacement and silent sites with a frequency between *u* and *v*, then

$$\sigma_{\text{u},\text{v}}=\sum_{\text{i}=1}^{\text{K}}{\text{s}}_{\text{i}}.\text{P}(\text{u}<{\text{p}}_{\text{i}}<\text{v}|\text{N},{\text{D}}_{\text{i}})$$ (4)

$$\rho_{\text{u},\text{v}}=\sum_{\text{i}=1}^{\text{K}}\left(1-{\text{s}}_{\text{i}}).\text{P(u}<{\text{p}}_{\text{i}}<\text{v}|\text{N},{\text{D}}_{\text{i}}\right)$$ (5)

Where *s*_i_ and (1-*s*_i_) represent the probabilities of a site being silent or replacement, respectively (see [26]). Thus, if the sampled sequences contain *S* silent sites and *R* replacement sites then *σ*_0,1_ = S, *ρ*_0,1_ = R and S+R = K.

### Estimating the number of adaptive sites

Following the theoretical and empirical analyses in [25], three site-frequency ranges are defined in this study: “low frequency” (0%-15%), “mid frequency” (15%-75%) and “high frequency” (75%-100%). The expected number of silent and replacement sites in each range were calculated using eqs 4 and 5. The expected number of silent sites in the low, mid and high frequency ranges are denoted σ_l_, σ_m_ and σ_h_, and the number of replacement sites in the same ranges are denoted ρ_l_, ρ_m_, and ρ_h_ (Fig 1C). If silent mutations and mid-frequency polymorphisms are selectively neutral, and deleterious mutations are confined to the low frequency range, then the expected number of adaptive sites (*α*_h_) can be estimated as:

$${\text{α}}_{\text{h}}=\rho_{\text{h}}\left(1-\frac{{\text{σ}}_{\text{h}}}{\rho_{\text{h}}}.\frac{\rho_{\text{m}}}{\sigma_{\text{m}}}\right)$$ (6)

This is identical to equation 1 in [25] except that the silent and replacement counts are estimated probabilistically using the Beta-Binomial sampling model (eqs 4 and 5). The number of adaptive sites can be converted to a per-codon rate of molecular adaptation by dividing by the number of codons in the sequence alignment and the time elapsed between the two sampling points (see Fig 1A). The assumptions on which the estimator in eq 6 is based were explored in [25] and appear to be robust for rapidly evolving viruses provided that viral effective population sizes are sufficiently large (> = 500). Recent estimates of the effective population size of HIV population within infected individuals (and which do not rely on a neutral coalescent model) are in the range of ~10^5^, strongly indicating that the molecular evolution of HIV is likely to be dominated by deterministic rather than stochastic forces [36].

The term in brackets in eq 6 represents an estimate of the fraction of replacement sites in the high frequency range that are driven by positive selection. The term $\frac{\rho_{\text{m}}}{\sigma_{\text{m}}}$ denotes the ratio of replacement-to-silent sites in the mid site-frequency range, which we refer to as the ‘neutral’ ratio and provides the baseline against which polymorphism in other site frequency ranges is compared. To assess statistical uncertainty a bootstrapping approach was undertaken using the procedure outlined in [25].

To verify that sequences generated after HMA screening are suitable for estimating adaptation rates, we analyzed a comparable within-host HIV-1 dataset that was generated without any HMA screening [5]. We emulated the effects of HMA-screening on this dataset by replacing all sets of sequences with >99% sequence identity by a single representative sequence. Site-frequencies and adaptation rates were then estimated from the original and screened datasets using the methods described above (S1 Fig). As expected, the number of low-frequency polymorphisms in the HMA-screened dataset was underestimated (S1A Fig). However, adaptation rates were similar between the two datasets (S1B Fig), likely because our method explicitly ignores low frequency sites. Although screening led to a slight underestimation of adaptation rate in a few patients (p1, p2, and p3) the effect is small compared to estimation error (S1B Fig) and therefore our results are qualitatively robust.

## Results and Discussion

**Fig 2:**
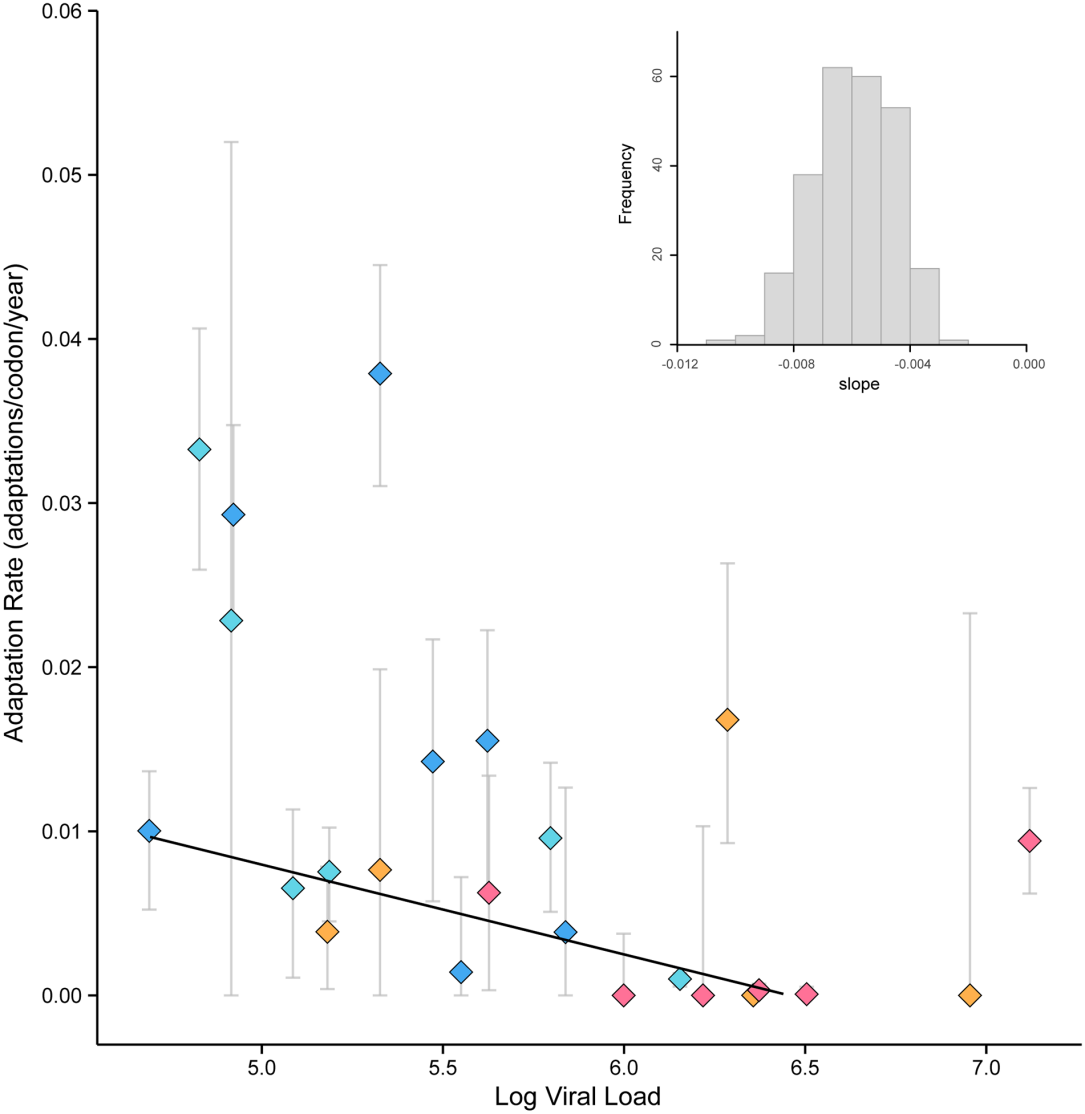
Viral adaptation rate is negatively correlated with viral population size in 24 pediatric HIV infections (Spearman’s rank correlation: p < 0.01). The trend-line was estimated using a weighted regression analysis (weighted regression: b = -0.0054, p < 0.01). The inset illustrates the bootstrap distribution of the slope, estimated from weighted regression, which indicates that the slope is less than zero. Data points are labeled by color according to the disease progression category of each patient as follows: slow non-progressors (SNP, dark blue), moderate non-progressors (MNP, light blue), moderate progressors (MP, orange), and rapid progressors (RP, pink). Error bars representing the uncertainty in our estimate were obtained using the bootstrap procedure described in [25]. Specifically, the error bars depict the lower and upper quartile estimates from 250 bootstrap samples.

We estimated absolute rates of HIV-1 molecular adaptation during 24 pediatric infections. Fig 2 presents the estimated rate of molecular adaptation for each patient, measured as the expected number of adaptive changes per codon per year. The estimated rates varied significantly among patients, ranging from >0.03 adaptations/codon/year in two patients to zero in six patients (mean among patients = 0.01 adaptations/codon/year). These rates are similar in magnitude to comparable estimates for nine adult HIV-1 infections, obtained from a 300 nt stretch of the C2-V3 region of the HIV-1 *env* gene [12]. When converted into the units used here, those adult infections averaged 0.029 ± 0.01 adaptations/codon/year (Table 2 in [12]). Rates of HIV-1 intra-host molecular adaptation appear faster than comparable estimates for the inter-host adaptation of human influenza A viruses, which do not exceed 0.008 adaptations/codon/year even in the fastest-adapting region of the influenza virus genome (i.e. the surface residues of the antigenic hemagglutinin protein [25]).

In Fig 2, for each patient, the estimated rate of viral adaptation is plotted against average log viral load. Viral load represents the number of virions circulating in each ml of peripheral blood and changes in viral load within an infected individual are proportional to and dependent on the number and productivity of actively infected cells [37]. Fig 2 demonstrates a striking negative correlation between rate of viral adaptation and log viral load (Spearman’s rank correlation; *r* = -0.620; *p*<0.01). The 95% percentile interval for the correlation coefficient from 250 bootstrap samples did not overlap with zero. A weighted regression analysis, in which weights correspond to the standard deviation of each estimate based on 250 bootstrap replicates, also indicates a statistically significant negative relationship in the empirical data (Student’s t-test; *b* = -0.0054; *p* < 0.01). Moreover, despite a comparatively small sample size, the entire bootstrap distribution of the regression slope is less than zero (Fig 2; inset). Importantly, this relationship is not sensitive to the threshold values used to define site-frequency ranges: the negative correlation remains significant (at the *p*<0.05 level) even when the mid-frequency range is redefined as 20%-80% or 10%-90% (see Methods).

The negative correlation in Fig 2 is both surprising and counterintuitive, as standard models of nucleotide fixation by positive selection predict the opposite relationship. Specifically, higher population sizes are expected to lead to greater adaptation rates, primarily because the rate at which new mutations are introduced into the population is linearly proportional to population size [38]. As a secondary effect, the fixation probability of weakly beneficial mutations may be higher due to a lessened role of random genetic drift when effective population sizes are larger [39, 40]. The viral adaptation rates in Fig 2 also show a strong association with disease progression. In particular, rates of viral adaptation are higher for patients defined as non-progressors (slow non-progressors, SNP, and moderate non-progressors, MNP), than those defined as progressors (moderate progressors, MP, and rapid progressors, RP). The mean adaptation rates in these two groups are 0.015 and 0.004 adaptations per codon per year, respectively, and are significantly different (Mann-Whitney *U*-test; *p* < 0.005). As expected, rapid progressors are characterized by higher viral loads and substantially lower CD4+ counts than non-progressors.

**Fig 3:**
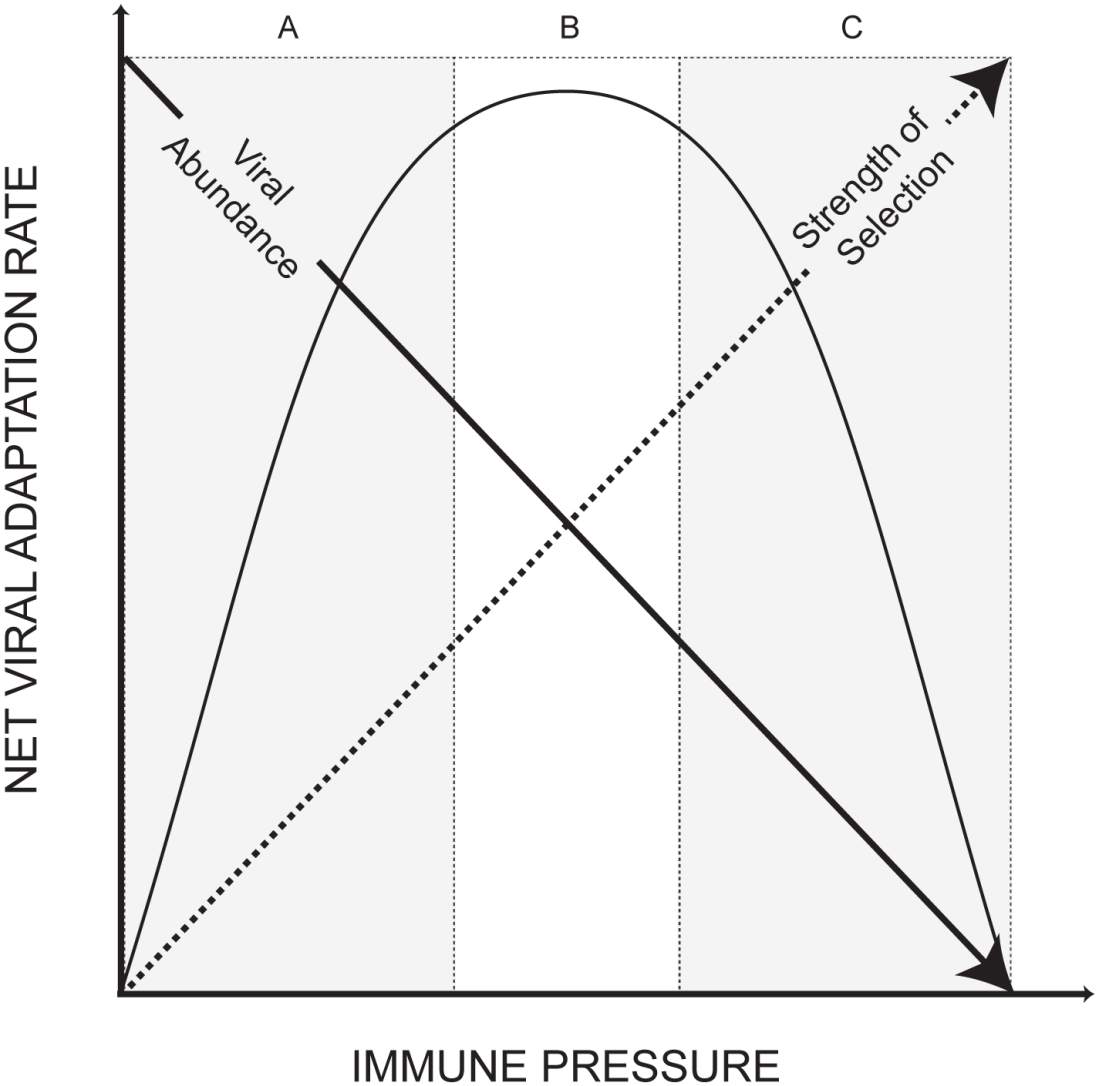
This figure is adapted from Fig 2a in Grenfell et al. [41]. A simple population genetics model predicts that absolute within-host viral adaptation rate varies non-linearly with host immune response, which has a opposing effects on viral population size and the strength of immune selection. The left-hand side of the curve (A) can explain the negative relationship observed in Fig 2: a weak immune response corresponds to large viral population but lower selective pressure. The shaded parts of the curve indicated by B and C predicts an absence or a positive relationship, respectively, between viral adaptation rate and viral population size.

The significant negative correlation between virus population size and adaptation rate observed here clearly requires explanation. Grenfell et al. [41] previously proposed that within-host viral adaptation rates might be non-linear with respect to the strength of the host immune response. Using a simple population genetic model they considered the opposing effects of host immune responses on viral population size and on the strength of selection imposed upon the viral population (Fig 3). Under this model, rates of adaptation are highest when immune responses are of intermediate strength. An important property of this model is that it predicts that the relationship between viral adaptation rate and viral population size can, under some circumstances, be negative, and can therefore explain the counterintuitive result in Fig 2. Specifically, the left-hand side (region A) of the plot in Fig 3 indicates that a negative correlation is expected when immune responses are comparatively weak, resulting in high viral loads and weak selective pressure. Crucially, this scenario is consistent with the clinical presentation of pediatric HIV infection, compared to adult infection. In other words, when the host-virus system is placed to the left of the peak adaptation rate, an increase in the potency of the immune response will result in an increase in the rate of viral adaptation, even though the viral population size is reduced and the rate of disease progression is correspondingly slowed. Conversely for adult HIV infections, where immune responses range from moderate to strong, we expect either no or a positive relationship between rate of viral adaptation and viral population size, i.e. the relationship is best explained by region B or C in Fig 3. Re-analysis of the adult HIV cohort from Shankarappa et al [5] supports this prediction since we find no association between viral adaptation rate and viral load (S3 Fig).

**Fig 4:**
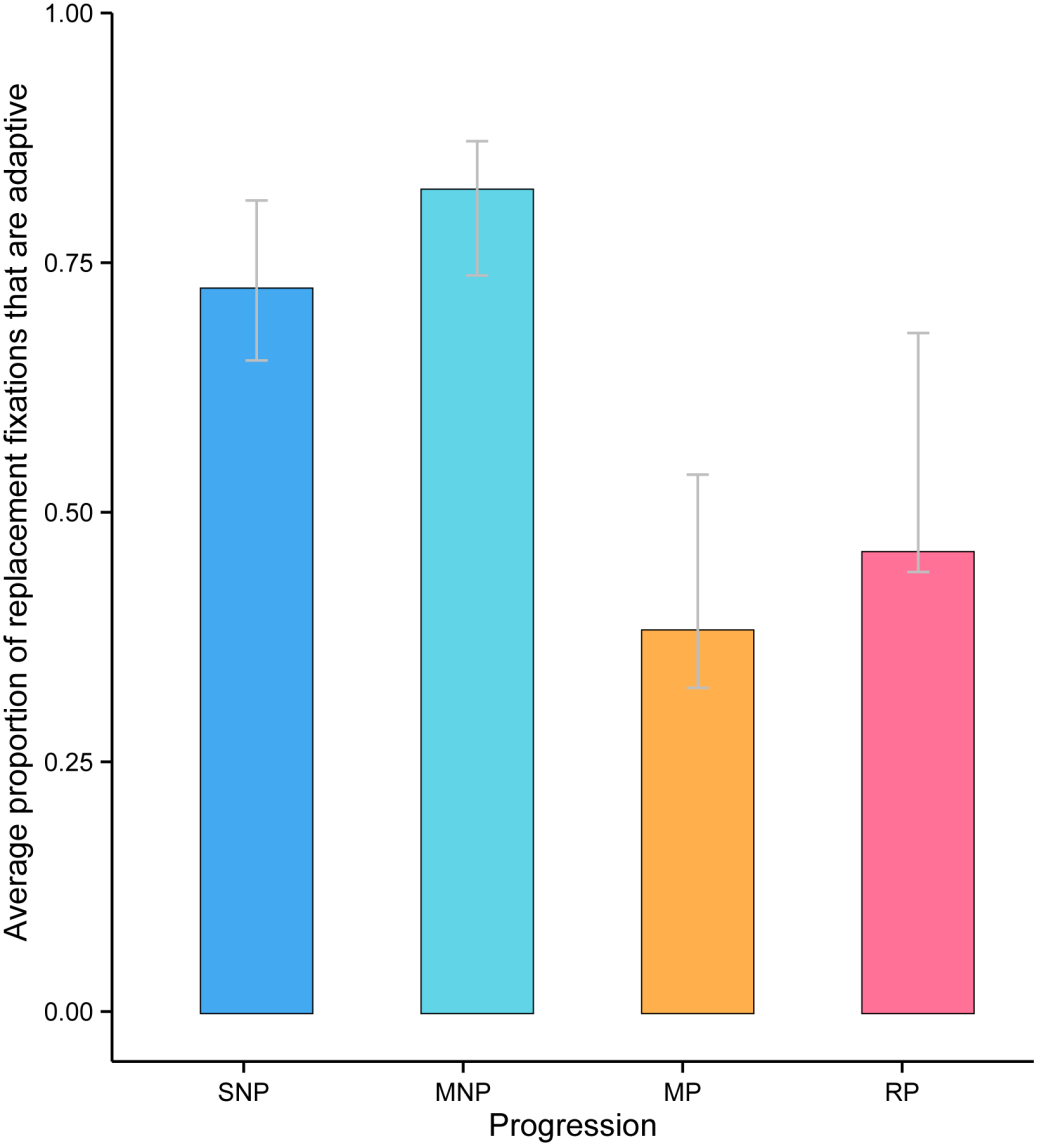
The proportion of high-frequency replacement polymorphisms that are adaptive (i.e. they have risen to high frequency due to positive selection) is significantly different greater in non-progressors (SNP and MNP) than progressors (MP and RP) (Mann-Whitney U-test, P < 0.05). This indicates that the strength of immune selection is significantly different among individuals with different disease outcomes. Error bars correspond to the interquartile range estimated from 250 bootstrap samples.

**Fig 5:**
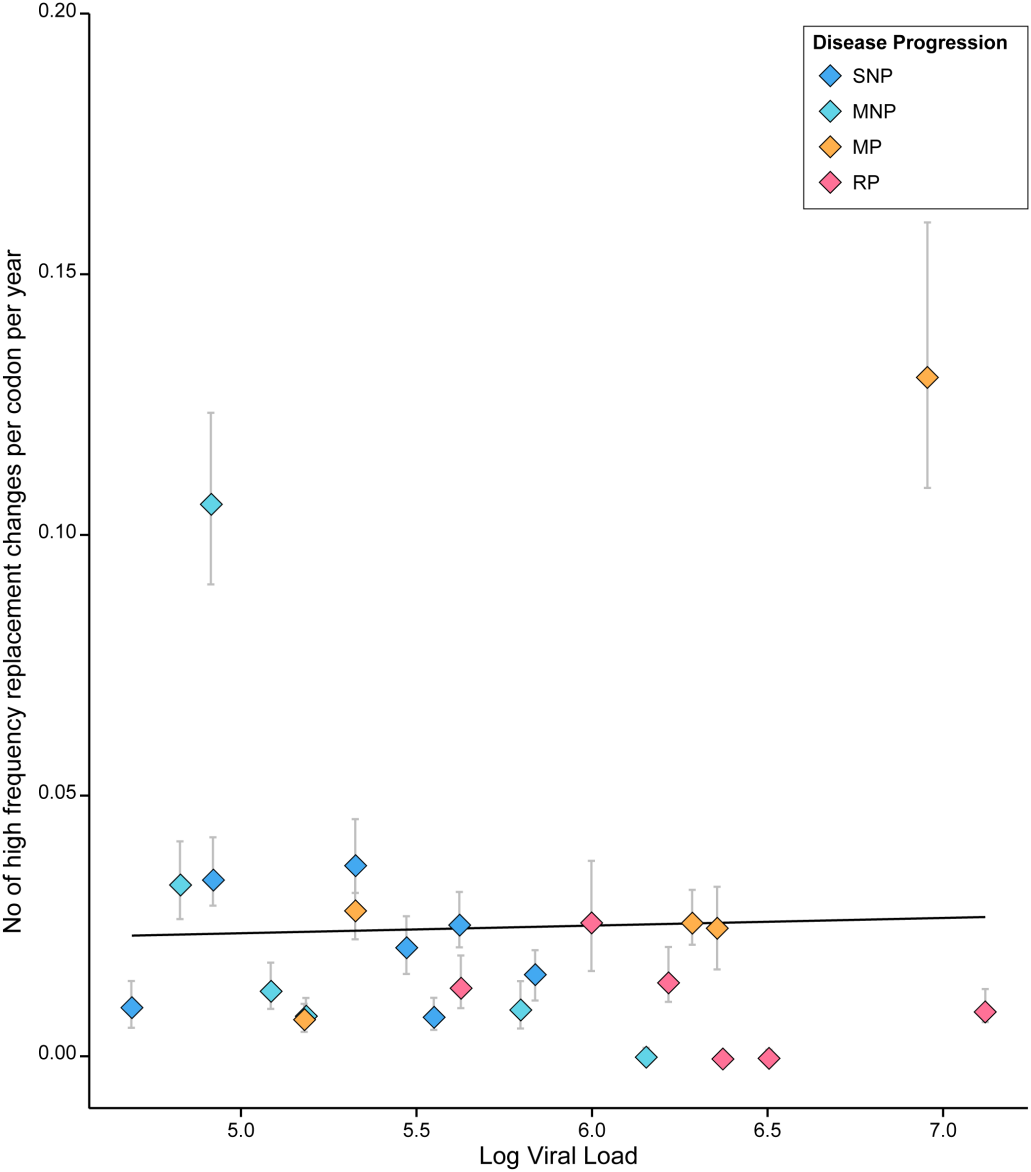
The number of high-frequency replacement polymorphisms (scaled by the number of codons in each alignment and the number of years of observation) is not correlated with viral population size (Spearman’s rank correlation; p > 0.05). If natural selection were weak compared to genetic drift then a negative correlation would be expected, due to an increased fixation of slightly deleterious mutations in populations of small size. The data points are labeled using the color scheme employed in Fig 2.

If the model in Fig 3 is correct, then it predicts the variation in adaptation rate seen in Fig 2 is primarily driven by variation in host immune response. Specifically, it proposes that viral adaptation is faster in patients who progress slower to disease because the selection coefficients of viral mutations are higher in those patients, and this increase outweighs the counteracting effect of smaller viral population size. To test this we calculated the proportion of high-frequency replacement polymorphisms that are adaptive, i.e. *α*_h_/*ρ*_h_, for each patient in the four different disease progression categories (Fig 4). This proportion is significantly greater for non-progressors than progressors (Fig 4; Mann-Whitney *U* test: *p*< 0.05), indicating that the observed variation in adaptation rate (Fig 2) is driven by a greater probability of fixation in patients with slower disease progression. To additionally test this interpretation, we plotted, for each patient, the total number of replacement polymorphisms (per codon per year) in the high site-frequency class (Fig 5). This represents the net rate of amino acid change, which is affected by both selection and genetic drift. This rate does not vary significantly among infected patients, despite the fact that viral population size ranges from 10^4^ to 10^7^ RNA copies per ml (Fig 5). This result is inconsistent with the hypothesis that the negative correlation observed in Fig 2 is the result of greater purifying selection when viral load is high. Therefore, although within-host viral population size varies greatly, it is nevertheless always sufficiently large in these patients for selection to be effective. Further, it seems unlikely that the negative relationship could be driven by variation in intrinsic mutations rates because we see no significant variation in the silent fixation rate in this cohort (S2c Fig). This observation also argues against strong selection on silent mutations in our study; such selection is not expected because (i) the region of *env* investigated does not include overlapping reading frames and (ii) immune-mediated selection on amino acid changes is likely to be overwhelmingly stronger. Further, we find no support for the hypothesis that the neutral ratio, $\frac{\rho_{\text{m}}}{\sigma_{\text{m}}}$, is positively correlated with viral load (S2a Fig).

We therefore conclude that the negative correlation between viral load and adaptation rate is primarily caused by the positive trend between $\frac{\sigma_{h}}{\rho_{h}}$ and viral load (S2b Fig). This is best explained by a change in the distribution of mutational selection coefficients engendered by immune selection. Specifically, stronger and/or faster humoral immune responses will increase the likelihood that replacement changes are advantageous, and this shift has a greater effect on net adaptation rate than the concomitant reduction in viral population size (as represented in Fig 3).

Lastly, to test the impact of the new Beta-binomial sampling prior approach, we re-analyzed our data using our previous approach [25], which does not include this sampling model (S4 Fig). The results were largely unchanged; in fact statistical support for a negative relationship between adaptation rate and viral population size very slightly increased when sampling error was ignored. This suggests that the Beta-binomial model more adequately reflects the additional estimation uncertainty arising from small sample sizes.

### Conclusions

Although the sets of virus gene sequences analyzed here are modest in size in comparison to some recent studies [42, 43], the cohort we analyzed is unusual in that (i) it represents pediatric rather than adult HIV-1 infections, (ii) informative data on viral load and the outcome of infection was available for each patient, and (iii) there were sufficient numbers of patients in each disease category to permit statistical comparison. We argue that two opposing consequences of immune selection on viral molecular evolution have led to an unexpected inverse relationship between the rate of viral adaptation and population size. Specifically, the counteracting effects of host immune responses upon viral abundance and viral selection coefficients can explain the pattern observed in Fig 2. This is the first time that the non-linear model of viral adaptation first formalized in Grenfell et al. [41] has been used to explain patterns in empirical data.

This study highlights the benefits of re-analyzing previously published data sets when new methods of analysis become available. For example, Carvajal-Rodriguez et al. [6] also investigated the same pediatric HIV cohort using dN/dS based methods, yet did not find a significant relationship between viral adaptation and disease progression. Measuring adaptive evolution by estimating the rate at which natural selection fixes beneficial mutations is complementary to, and has some advantages over, alternative approaches methods that estimate dN/dS ratios, or which estimate selection coefficients. First, the absolute rates of viral adaptation obtained here can be interpreted directly, whereas the correct interpretation of dN/dS ratios in the context of within-host virus evolution is uncertain [10]. Per-year adaptation rates are directly comparable among different populations and even among species. Second, estimation of mutational selection coefficients often requires parametric population genetic models that make strong assumptions about population demography or the mode of selection [44, 45], which, if not correct, could lead to misleading results. In contrast, net rates of viral adaptation can be estimated without making any assumptions about the population and selection dynamics in the studied population, which may be very complex for rapidly evolving viruses including HIV-1 [46]. Third, even when such values can be estimated, their general relevance is unclear because fitness and selection coefficients are typically defined relative to the environment in which they are measured. The virus’ immune environment will vary significantly, both among hosts and through time within each host, making quantitative comparison of selection coefficients very difficult except under highly controlled experimental conditions, such as growth in cell culture [47]. However, it is important to note that the site frequency-based approach used here cannot identify the specific codons that are under positive selection and therefore other methods, such as dN/dS, should be used when that is the question of interest.

Advances in HIV treatment necessitate the re-analysis of published HIV data in order to understand the virus’ evolutionary behavior, even when those data were generated using older sequencing techniques such as HMA screening. Modern highly active anti-retroviral therapy (HAART) reduces viremia to low or undetectable levels in most cases and it would be unethical to recruit new cohorts of chronically infected patient without providing them with treatment. Consequently, data sets that predate the widespread use of HAART, such as the pediatric cohort analyzed here, provide an irreplaceable source of information about the natural ecology and evolution of HIV during infection.

The negative relationship between net viral adaptation rate and population size discovered here has consequences for the interpretation and prediction of the outcome of pediatric HIV-1 infection. Specifically, if childhood HIV infections do indeed lie on the left hand side of the model in Fig 3, then potential interventions that aim to boost humoral “immune responses” will likely lead to an increased, not decreased, rate of viral adaptation, despite generating a lower viral load. This could mean that the benefits of any such intervention are short lived, unless the intervention itself can adapt on the same timescale as the viral population. Lastly, the model shown in Fig 3 suggests that adult HIV infections should be further towards the right (regions B and C), as immune responses are stronger on average than for pediatric infection. As a consequence, we predict either no relationship, or a positive one, between rate of viral adaptation and viral load for adult HIV-1 infection. Our re-analysis of the adult HIV cohort from Shankarappa et al [5] matches this prediction because it shows no association between viral adaptation rate and viral load among patients (S3 Fig). However, the results in S3 Fig should be interpreted with caution because that cohort contains far fewer patients and the range of viral load values is narrower, both of which will act to reduce statistical power to detect a trend. It has also been previously noted that there is a positive relationship between adaptation rate and disease progression in HCV infections [10]. This indicates that variation in within-host adaptation rates in HCV are most likely explained by region C in Fig 3, where increasing immune selection on the viral population could lead to clearance of the infection.

Understanding the effects of different host immune responses on HIV evolution during infection is also important to vaccine design and treatment. Since historic (i.e. pre-HAART) data sets mostly represent HIV *env* sequences, it is difficult to investigate viral adaptation in other genomic regions. Thus our study was restricted to examining how variability in *env* adaptation rates among patients is explained by viral load. However, it is clear that viral adaptation outside of *env* (e.g. escape from CTL immune responses [48]) is important in determining variation in viral replication and disease progression. Therefore our results should *not* be used to support the inverse argument, i.e. that *env* adaptation rates explain variation in viral load. In order to fully understand the relative importance of different host immune responses in shaping viral load additional sequence data that represent genes other than *env* is required.

## Supporting Information

S1 Fig The effects of HMA-screening method are tested on a canonical within-host HIV dataset from Shankarappa et al [5]. A HMA-screened dataset was generated as described in Methods. The results for HMA screened and full dataset are represented in blue and red respectively. The error bars indicate the uncertainty in derived site-frequency estimate due to the ancestral alignment (i.e. site-frequencies were re-estimated using each sequence in the ancestral alignment). In the HMA-screened datasets, there is a tendency for the number of low-frequency polymorphisms (pandel A) to be underestimated. Although this can lead to an underestimation of the adaptation rate in some cases the relationship of the estimated adaptation rates between HMA-screened (x-axis) and full (y-axis) datasets (panel B) show good agreement. The dashed grey line indicates x = y line.
(TIF)

S2 FigA) Neutral Ratio ($\frac{\rho_{m}}{\sigma_{m}}$) versus viral load. The observation of silent to replacement polymorphisms at intermediate site-frequency is not strongly correlated with viral load suggesting the mutation rates are largely similar among the 24 patients. B) The ratio of silent and replacement polymorphisms in the high site-frequency class ($\frac{\sigma_{h}}{\rho_{h}}$) versus viral load. The positive trend observed suggests that the rate at which high-frequency replacement polymorphisms appear relative to silent polymorphisms in the population decreases with increasing viral loads. C) The relationship between silent fixation rate (calculated as the number of high-frequency silent polymorphisms per codon per year) against viral load. There is no significant variation among patients and supports that mutation rates of the within-host HIV populations are not substantially different among patients.(TIF)

S3 Fig The relationship between adaptation rate and viral load in adult HIV infections (based on the Shankarappa cohort) is shown. This still supports the non-linear model of adaptation where immune responses vary from weak to strong, as either no distinct or a positive trend is expected.
(TIF)

S4 Fig The re-analysis of the childhood HIV cohort without the Beta-binomial sampling prior supports a negative relationship between viral adaptation rate and viral population size with the bootstrap distribution of Spearman ‘r’ excluding zero. (TIF)

S1 Table Clinical information for all 24 patients. (DOCX)

S2 Table Assuming equal codon frequencies and an equal transition/transversion ratio, codon positions where any nucleotide mutation results either a silent or replacement change are assigned 1 and 0, respectively. Codon positions where silent and replacement change are possible are assigned 0.5. As a consequence, the probability of observing a silent mutation, p(s), at the three codon positions are 0.0625, 0.0, and 0.711. The probability of observing a replacement mutation is just 1-p(s), i.e. 0.9375, 1.0, and 0.289.
(DOCX)
