# Supplementary figures and images for: Faster Adaptation in Smaller Populations: Counterintuitive Evolution of HIV during Childhood Infection
Source: PLoS Comput Biol. 2016 Jan 7;12(1):e1004694. doi: 10.1371/journal.pcbi.1004694 (PMC4704780; doi:10.1371/journal.pcbi.1004694)

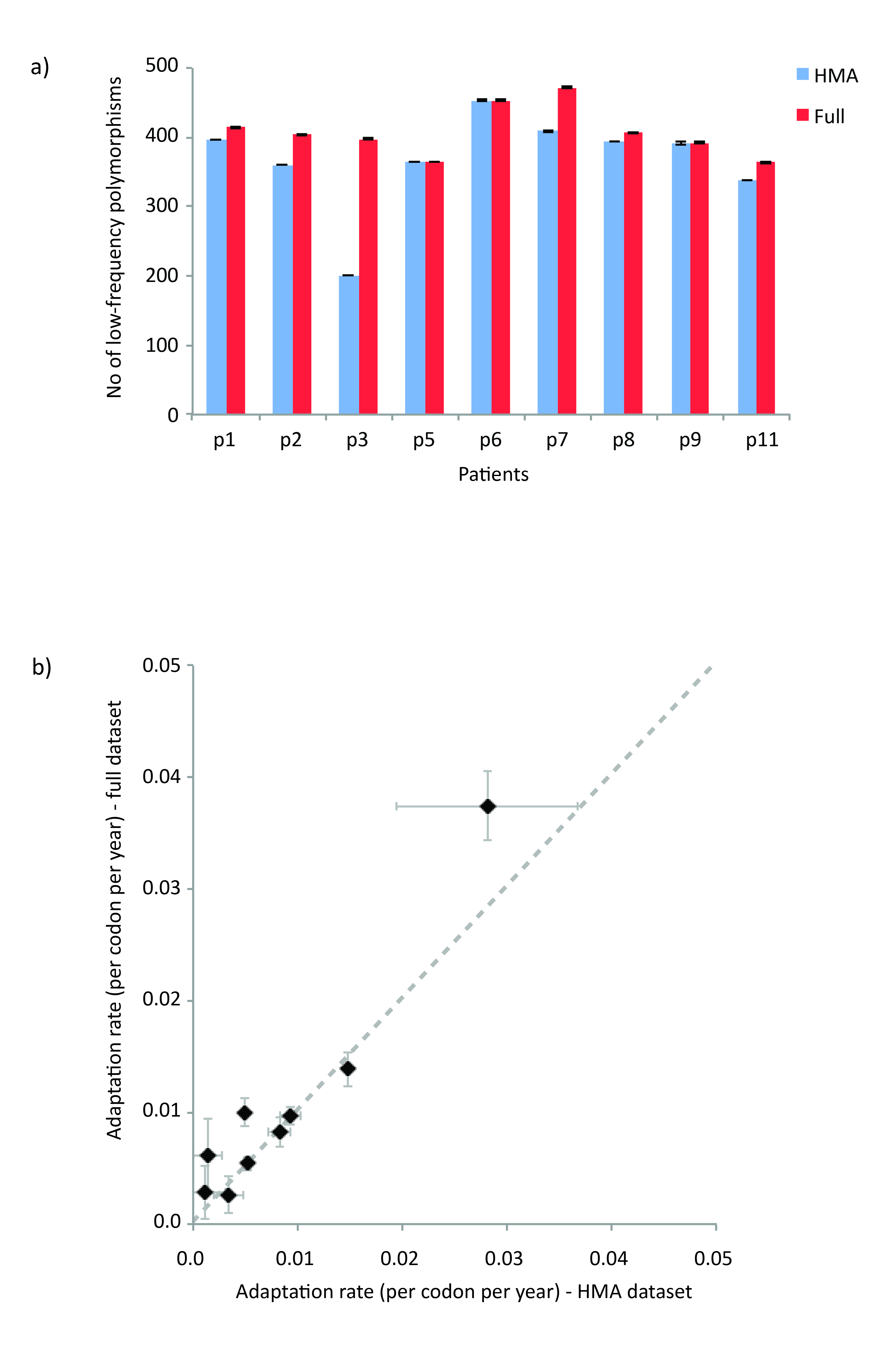

Supplement: S1 Fig — A HMA-screened dataset was generated as described in Methods. The results for HMA screened and full dataset are represented in blue and red respectively. The error bars indicate the uncertainty in derived site-frequency estimate due to the ancestral alignment (i.e. site-frequencies were re-estimated using each sequence in the ancestral alignment). In the HMA-screened datasets, there is a tendency for the number of low-frequency polymorphisms (pandel A) to be underestimated. Although this can lead to an underestimation of the adaptation rate in some cases the relationship of the estimated adaptation rates between HMA-screened (x-axis) and full (y-axis) datasets (panel B) show good agreement. The dashed grey line indicates x = y line. (TIF) [file pcbi.1004694.s001.tif]

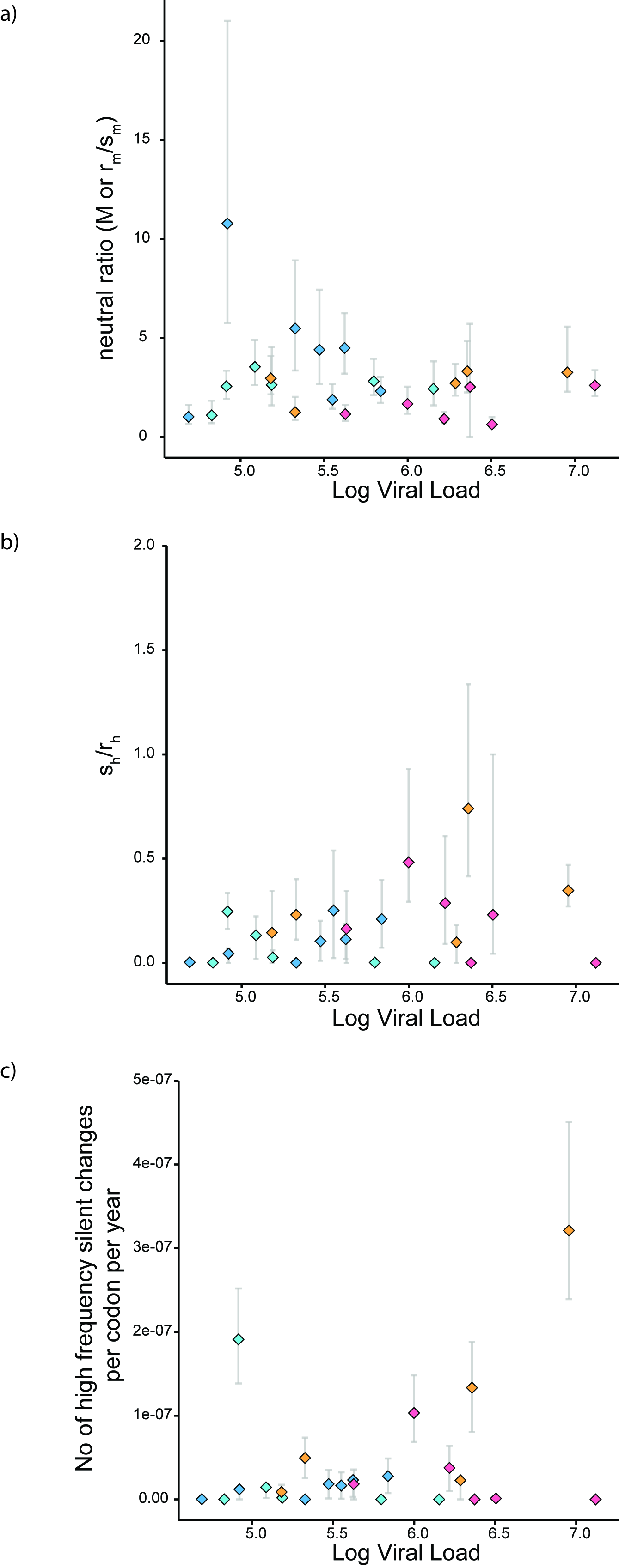

Supplement: S2 Fig — A) Neutral Ratio (ρmσm) versus viral load. The observation of silent to replacement polymorphisms at intermediate site-frequency is not strongly correlated with viral load suggesting the mutation rates are largely similar among the 24 patients. B) The ratio of silent and replacement polymorphisms in the high site-frequency class (σhρh) versus viral load. The positive trend observed suggests that the rate at which high-frequency replacement polymorphisms appear relative to silent polymorphisms in the population decreases with increasing viral loads. C) The relationship between silent fixation rate (calculated as the number of high-frequency silent polymorphisms per codon per year) against viral load. There is no significant variation among patients and supports that mutation rates of the within-host HIV populations are not substantially different among patients. (TIF) [file pcbi.1004694.s002.tif]

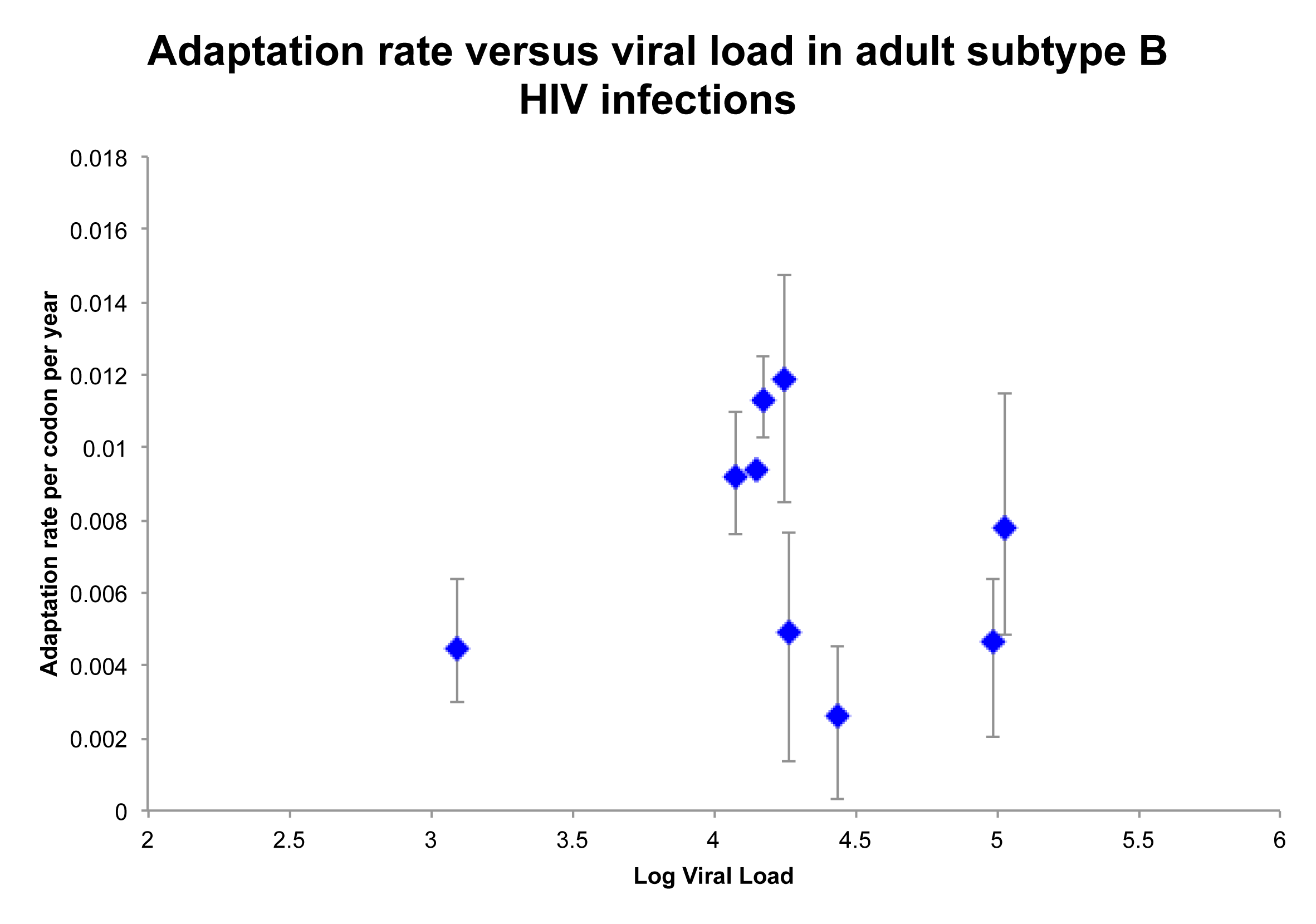

Supplement: S3 Fig — This still supports the non-linear model of adaptation where immune responses vary from weak to strong, as either no distinct or a positive trend is expected. (TIF) [file pcbi.1004694.s003.tif]

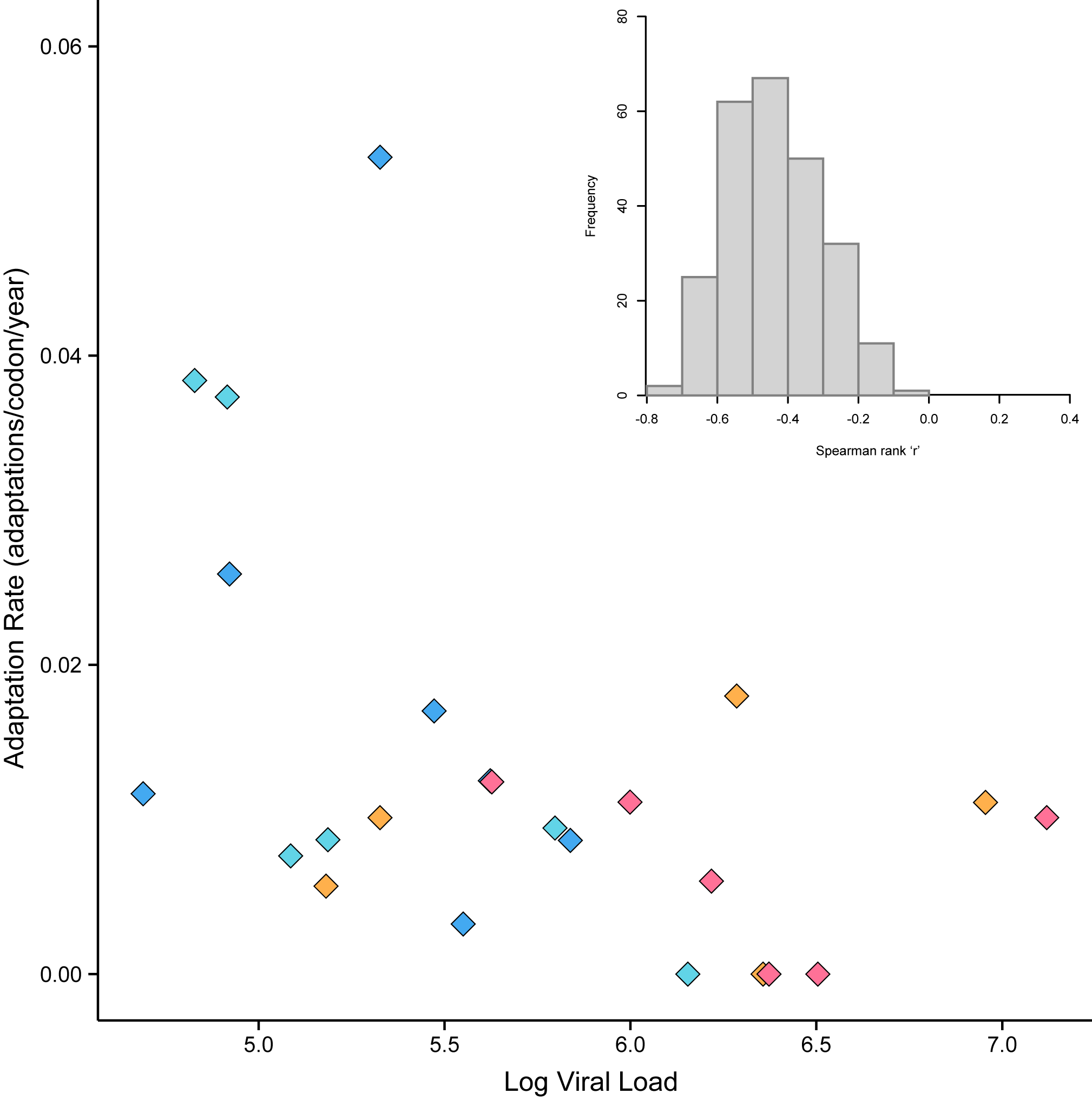

Supplement: S4 Fig — (TIF) [file pcbi.1004694.s004.tif]
