# Supplementary material for: Faster Adaptation in Smaller Populations: Counterintuitive Evolution of HIV during Childhood Infection
Source: PLoS Comput Biol. 2016 Jan 7;12(1):e1004694. doi: 10.1371/journal.pcbi.1004694 (PMC4704780; doi:10.1371/journal.pcbi.1004694)
Supplement: S1 Table — (DOCX) [file pcbi.1004694.s005.docx]

**Table S1: Patient characteristics**

| **Patient** | **Clinical prognosis ^a^** | **Anti-retroviral therapy ^b^** | **Mean viral load (log RNA copies/ml)** | **Average CD4+ T-cell count** |
| --- | --- | --- | --- | --- |
| p1 | MNP |  | 5.80 | 1220.06 |
| p2 | MP | AZT | 6.36 | 1616.66 |
| p3 | SNP |  | 5.33 | 3867.35 |
| p4 | MNP | AZT/DDI | 6.15 | 3726.11 |
| p5 | MNP | AZT | 4.92 | 1105.50 |
| p6 | MNP |  | 4.83 | 1488.73 |
| p7 | SNP |  | 4.92 | 1522.16 |
| p8 | MP | AZT/DDI | 5.33 | 2271.12 |
| p9 | MNP | AZT | 5.19 | 1717.16 |
| p10 | MP | AZT | 6.29 | 845.81 |
| p11 | SNP | AZT/DDI | 5.55 | 3540.79 |
| p12 | SNP |  | 5.47 | 2451.63 |
| p13 | MP | AZT/DDI | 5.18 | 1802.46 |
| p14 | MNP | AZT | 5.09 | 896.91 |
| p15 | SNP |  | 5.62 | 2054.50 |
| p16 | SNP |  | 5.84 | 3442.45 |
| p18 | RP | AZT | 6.00 | 1346.85 |
| p19 | SNP | AZT | 4.69 | 2340.10 |
| p20 | RP | AZT | 6.37 | 490.55 |
| p21 | RP | AZT/DDI | 7.12 | 470.74 |
| p22 | RP | AZT/DDI | 6.50 | 341.46 |
| p23 | RP | AZT/DDI | 5.63 | 1294.84 |
| p24 | RP | AZT/DDI | 6.22 | 791.26 |
| p25 | MP |  | 6.96 | 2493.63 |

^a^ Prognosis was ascertained from CD4+ T-cell count and clinical diagnosis of AIDS. For further details see Edwards et al (20)

^b^ AZT=Azidothymidine; DDI=dideoxyinosine. For further details see supplementary information from Carvajal-Rodriguez et al (6)
