# Supplementary material for: Faster Adaptation in Smaller Populations: Counterintuitive Evolution of HIV during Childhood Infection
Source: PLoS Comput Biol. 2016 Jan 7;12(1):e1004694. doi: 10.1371/journal.pcbi.1004694 (PMC4704780; doi:10.1371/journal.pcbi.1004694)
Supplement: S2 Table — Codon positions where silent and replacement change are possible are assigned 0.5. As a consequence, the probability of observing a silent mutation, p(s), at the three codon positions are 0.0625, 0.0, and 0.711. The probability of observing a replacement mutation is just 1-p(s), i.e. 0.9375, 1.0, and 0.289. (DOCX) [file pcbi.1004694.s006.docx]

**Table S2: Codon degeneracy table and the relative probabilities of observing a silent mutation at each codon position.**

| **Amino Acid** | **Codon** | **Pos1** | **Pos2** | **Pos3** | **Amino Acid** | **Codon** | **Pos1** | **Pos2** | **Pos3** |
| --- | --- | --- | --- | --- | --- | --- | --- | --- | --- |
| Lysine | AAA | 0 | 0 | 0.5 | Glutamate | GAA | 0 | 0 | 0.5 |
| Asparagine | AAC | 0 | 0 | 0.5 | Aspartic acid | GAC | 0 | 0 | 0.5 |
| Lysine | AAG | 0 | 0 | 0.5 | Glutamate | GAG | 0 | 0 | 0.5 |
| Asparagine | AAT | 0 | 0 | 0.5 | Aspartic acid | GAT | 0 | 0 | 0.5 |
| Threonine | ACA | 0 | 0 | 1 | Alanine | GCA | 0 | 0 | 1 |
| Threonine | ACC | 0 | 0 | 1 | Alanine | GCC | 0 | 0 | 1 |
| Threonine | ACG | 0 | 0 | 1 | Alanine | GCG | 0 | 0 | 1 |
| Threonine | ACT | 0 | 0 | 1 | Alanine | GCT | 0 | 0 | 1 |
| Arginine | AGA | 0.5 | 0 | 0.5 | Glycine | GGA | 0 | 0 | 1 |
| Serine | AGC | 0 | 0 | 0.5 | Glycine | GGC | 0 | 0 | 1 |
| Arginine | AGG | 0.5 | 0 | 0.5 | Glycine | GGG | 0 | 0 | 1 |
| Serine | AGT | 0 | 0 | 0.5 | Glycine | GGT | 0 | 0 | 1 |
| Isoleucine | ATA | 0 | 0 | 0.5 | Valine | GTA | 0 | 0 | 1 |
| Isoleucine | ATC | 0 | 0 | 0.5 | Valine | GTC | 0 | 0 | 1 |
| Methionine | ATG | 0 | 0 | 0 | Valine | GTG | 0 | 0 | 1 |
| Isoleucine | ATT | 0 | 0 | 0.5 | Valine | GTT | 0 | 0 | 1 |
| Glutamine | CAA | 0 | 0 | 0.5 | Stop | TAA | 0 | 0 | 0 |
| Histidine | CAC | 0 | 0 | 0.5 | Tyrosine | TAC | 0 | 0 | 0.5 |
| Glutamate | CAG | 0 | 0 | 0.5 | Stop | TAG | 0 | 0 | 0 |
| Histidine | CAT | 0 | 0 | 0.5 | Tyrosine | TAT | 0 | 0 | 0.5 |
| Proline | CCA | 0 | 0 | 1 | Serine | TCA | 0 | 0 | 1 |
| Proline | CCC | 0 | 0 | 1 | Serine | TCC | 0 | 0 | 1 |
| Proline | CCG | 0 | 0 | 1 | Serine | TCG | 0 | 0 | 1 |
| Proline | CCT | 0 | 0 | 1 | Serine | TCT | 0 | 0 | 1 |
| Arginine | CGA | 0.5 | 0 | 1 | Stop | TGA | 0 | 0 | 0 |
| Arginine | CGC | 0 | 0 | 1 | Cysteine | TGC | 0 | 0 | 0.5 |
| Arginine | CGG | 0.5 | 0 | 1 | Tryptophan | TGG | 0 | 0 | 0 |
| Arginine | CGT | 0 | 0 | 1 | Cysteine | TGT | 0 | 0 | 0.5 |
| Leucine | CTA | 0.5 | 0 | 1 | Leucine | TTA | 0.5 | 0 | 0.5 |
| Leucine | CTC | 0 | 0 | 1 | Phenylalanine | TTC | 0 | 0 | 0.5 |
| Leucine | CTG | 0.5 | 0 | 1 | Leucine | TTG | 0.5 | 0 | 0.5 |
| Leucine | CTT | 0 | 0 | 1 | Phenylalanine | TTT | 0 | 0 | 0.5 |
